# Tonsillitis—Acute and Chronic*Read at meeting Austin District Medical Society, March 28, 1899.

**Published:** 1899-04

**Authors:** 


For Texas Medical Journal.

### Tonsillitis—Acute and Chronic.*

This is a subject over which there seems to be quite a difference of opinion, especially as to what period of life is most susceptible to the disease; some authors claim it to be a disease of adult life, while others claim that it occurs most frequently in childhood.

I am quite sure in this climate it occurs most frequently in children from two to seven years of age. I shall not undertake to describe all the varieties of tonsillitis, but will confine my paper to the form I see most frequently in practice; for to take up all the varieties would require considerable time.

Acute tonsillitis is an inflammation of the mucous membrane, and sometimes of the parenchyma of the tonsils, and may end in resolution, suppuration, or chronic enlargement. In the superficial variety, the surface is red and swollen, and is sometimes covered with a film of muco-pus, and is attended with fever and a sense of burning in the throat. In follicular tonsillitis, the lacunæ are filled with a cheesy mass of pus, and, sometimes, of particles of food, which have lodged in the folds of mucous membrane, which protrude from the tonsillar crypts. The follicles may form an abscess by breaking into each other, or they may form ulcers in the same manner, or rather by a process of necrosis. Parenchymatous tonsillitis is shown by greater enlargement of the tonsils due to a

*Read at meeting Austin District Medical Society, March 28, 1899.

marked infiltration of all the tissues, and may become so enlarged as to prevent deglutination.

Chronic tonsillitis often follows acute attacks, and is quite a common disease. It is quite common in subjects of the rheumatic diathesis. It often seems to run in families for generations, and is not connected with tuberculosis. Chappell, of New York, found in routine examination of two thousand children, that two hundred and seventy had enlarged tonsils. I believe that a larger per cent. than that quoted by Chappell exists in this country. Although I have not a tabulated report, it seems to me that nearly half of the children I examine have enlarged tonsils, due, I believe, to the prevalence of catarrh.

Symptoms.—Acute tonsillitis is usually of sudden onset, beginning with a chill, or chilliness, followed by fever and headache, and often, a general aching. Sometimes spasm may usher in the disease. The fever reaches its height in a few hours; there is often nausea, and vomiting; the pulse is rapid, full and bounding; the face is flushed.

Treatment.—The first and most important step in treatment is the free opening of the bowels, for which there is nothing better than small and often repeated doses of calomel, and the administration of drugs to reduce fever, and make the patient comfortable. For this there is nothing that acts better than phenacetine. Then the thorough cleansing and disinfection of the throat, to prevent noxious materials from being swallowed. Where there exists a rheumatic diathesis, the giving of anti-rheumatics is indicated; for which Holt recommends salol.

Some authors recommend colchicum, others guiaicum, while others believe in the salicylates.

